# Telephone coaching of carers: comparison with collaborative care skills workshop

**DOI:** 10.1186/2050-2974-1-S1-O20

**Published:** 2013-11-14

**Authors:** Ross King, Genevieve Pepin, Amy Brown, Amanda Dando

**Affiliations:** 1 Deakin University

## 

Caring for an individual with an eating disorder is associated with significant burden. Treasure and associates' Collaborative Care Skills Workshops reduce carer burden and distress through altering maladaptive expressed emotion communication patterns and teaching basic motivational interviewing skills which assist in boosting readiness to change in their loved one. However, due to time or distance constraints, the workshops are not accessible to all carers. The current study examined the effectiveness of a telephone coaching intervention, incorporating use of a self-help book and DVDs. Carers received four 40 minute coaching sessions delivered at fortnightly to three week intervals. Carer distress, coping patterns, expressed emotion, accommodation and enabling behaviours and perceived impact of the eating disorder on the carers were assessed pre-, post and six weeks' follow-up. Fifteen carers completed questionnaires at all three time points and were matched to 15 attendees of the carer workshop intervention. At follow-up, carers in the interventions showed similar reductions in psychological distress, perceived burden, emotional over-involvement, accommodation and enabling and maladaptive coping. Changes in adaptive coping and critical comments were not observed. Therefore, despite small participant numbers, the telephone intervention was comparable in impact, providing an effective alternate mode of delivery for carer interventions.

This abstract was presented in the **Care in Inpatient and Community Settings** stream of the 2013 ANZAED Conference.

